# Fuch’s Endothelial Corneal Dystrophy in Cataract Patients Is Associated with Elevated Levels of Inflammatory Chemokines, but Not Growth Factors, in the Aqueous Humor

**DOI:** 10.3390/ijms25031894

**Published:** 2024-02-04

**Authors:** Rafał Fiolka, Edward Wylęgała, Michał Toborek, Dominika Szkodny, Zenon Czuba, Adam Wylęgała

**Affiliations:** 1 Department of Ophthalmology, Faculty of Medical Sciences in Zabrze, Medical University of Silesia, 40-760 Katowice, Poland; ewylegala@sum.edu.pl (E.W.); dominikacholewa1@gmail.com (D.S.); 2 Doctoral School of the Medical University of Silesia in Katowice, 40-055 Katowice, Poland; 3 Department of Biochemistry and Molecular Biology, University of Miami School of Medicine, Miami, FL 33136, USA; mtoborek@med.miami.edu; 4 Department of Microbiology and Immunology, Faculty of Medical Science in Zabrze, Medical University of Silesia, 40-055 Katowice, Poland; zczuba@sum.edu.pl; 5 Health Promotion and Obesity Management, Pathophysiology Department, Medical University of Silesia in Katowice, 40-752 Katowice, Poland; adam.wylegala@gmail.com

**Keywords:** cataract, eotaxin, Fuch’s endothelial corneal dystrophy, IP-10, RANTES, VEGF

## Abstract

The study investigated a profile of chemokines and growth factors in the aqueous humor (AH) of eyes with Fuch’s endothelial corneal dystrophy (FECD) and cataracts in comparison with cataract patients as a control group. A total of 52 AH samples (26 FECD + cataract and 26 cataract/control) were collected before cataract surgery. None of the patients had any clinically apparent inflammation at the time of AH collection. The AH levels of MCP-1 (CCL2), MIP-1α (CCL3), MIP-1β(CCL4), RANTES (CCL5), eotaxin (CCL11), IP-10 (CXCL10), FGF basic, G-CSF, GM-CSF, PDGF-bb, and VEGF were compared between the groups. The analyses were performed using the Bio-Plex 200 System from Bio-Rad. Among the studied parameters, the AH levels of RANTES, eotaxin, and IP-10 significantly increased in the FECD + cataract eyes, compared with the cataract controls (*p* < 0.05). Elevated levels of the RANTES, Eotaxin, and IP-10 indicate more intense inflammation in the eyes of patients in the FECD + cataract group. Moreover, these factors exhibit potential as predictive biomarkers for early detection of FECD in cataract patients. The discovery of elevated concentrations of biochemical markers in a patient, who has not yet received a clinical diagnosis, may suggest the need for heightened observation of the other eye to monitor the potential development of FECD.

## 1. Introduction

Fuch’s endothelial corneal dystrophy (FECD) is a common bilateral and progressive disease of the endothelial layer of the cornea, which usually manifests in women. It is a degenerative disease characterized by corneal endothelial decompensation. FECD causes corneal stromal and epithelial edema and progressively develops into bullous keratopathy, which can eventually lead to blindness. It exists in two forms: rare early-onset and late-onset forms. The early-onset form is familial and presents from birth or early childhood and reaches advanced stages between the ages of 10 and 20 years. Late-onset FECD is the most common form, and its onset occurs typically after 40 years of age. Although many facts have been revealed about the genetic and molecular biology of FECD, the exact pathogenesis of this disease is still unknown [1,2]. Presently, around 300 million individuals aged 30 years old and above are believed to be affected by FECD worldwide [3]. 

FECD is often accompanied by an increased risk of primary open-angle glaucoma or cataracts [2]. A cataract is a lens abnormality characterized by decreased transparency and increased cloudiness. It is the leading cause of blindness and visual impairment globally, affecting ~16 million people worldwide [4,5,6]. Modification, aggregation, and precipitation of crystallins (the main proteins that make up the lens and lens surfaces, responsible for their refractive function) are the main mechanisms underlying cataract development. The most common form of cataract is the age-related cataract, which usually develops in individuals over age 50 [7]. In addition to increasing age and genetics, several risk factors for cataract have been identified, such as exposure to ultraviolet light, diabetes, or long-term use of corticosteroids [6]. Additionally, FECD could be another factor that influences cataract development. 

The cause of cataract development in individuals with FECD remains unclear. The only form of treatment for cataracts is surgery. However, cataract surgery in FECD eyes requires specific protection of the corneal endothelium. 

Regardless of the presence of cataract, it appears clinically interesting to determine the predictive factors in the development of FECD, especially in the first stage of FECD development. The discovery of elevated concentrations of biochemical markers in a patient, who has not yet received a clinical diagnosis, may suggest the need for heightened observation of the other eye to monitor the potential development of FECD. One potential group of such biomarkers can be based on a cytokine profile in the aqueous humor (AH).

In fact, alterations of cytokine composition in the aqueous humor have been observed in various ocular diseases; however, there is a lack of information about its profile in FECD and/or cataracts.

In the present study, we focused on the assessment of selected chemokines mostly from the CC (beta) group, namely, monocyte chemotactic protein (MCP-1/CCL2), macrophage inflammatory protein (MIP-1 alpha/CCL3 and MIP-1 beta/CCL4), Regulated on Activation, Normal T-cell Expressed and Secreted (RANTES/CCL5), Interferon-gamma (IFN-gamma)-inducible protein-10 (IP-10, CXCL10), and eosinophil-specific chemoattractant, eotaxin (CCL11), as well as growth factors such as VEGF, FGF basic, G-CSF, and PDGF-bb. These factors have been shown to be involved in the development of several ocular pathologies such as age-related macular degeneration, glaucoma, proliferative retinopathies, neovascularization, and uveitis [8,9,10]. Therefore, we hypothesized that they can also play a role in the pathogenesis of FECD. Hence, the aim of our study was to evaluate the chemokine and growth factor profile in the AH of patients diagnosed with FECD and cataract in comparison with cataract eyes, the control group.

## 2. Results

Table 1 presents summary statistics of statistically significant key ophthalmological variables measured in patients with a specific eye condition with *p*-values between the groups. Distribution between females and males was non-significant (*p* = 0.09). There were no differences in the age of patients between the groups. The median visual acuity was 0.180 (range: 0.0200 to 1.000) and the median intraocular pressure was 15.00 mmHg (range: 11.00 to 20.00).

The median spherical refractive error was 1.00 D (range: −14.00 to 7.00), while the median cylindrical refractive error was −0.75 D (range: −4.00 to 0.00), and the median axis was 70.0 (range: 0.00 to 179.00). The median corneal endothelial cell density was 2404.0 (range: 886.00 to 3331.00), and the median of the keratometry flat (K1) value was 43.89 (range: 29.51 to 59.80). The median axis of the K1 value was 75.0 (range: 0.00 to 179.00), and the median keratometry steep value (K2) was 44.54 D (range: 15.68 to 65.70). The axis value was 92.0 (range: 90.00 to 174.00). The median cylindrical power was −0.80 D (range: −18.1 to 0.00).

**Figure 1:**
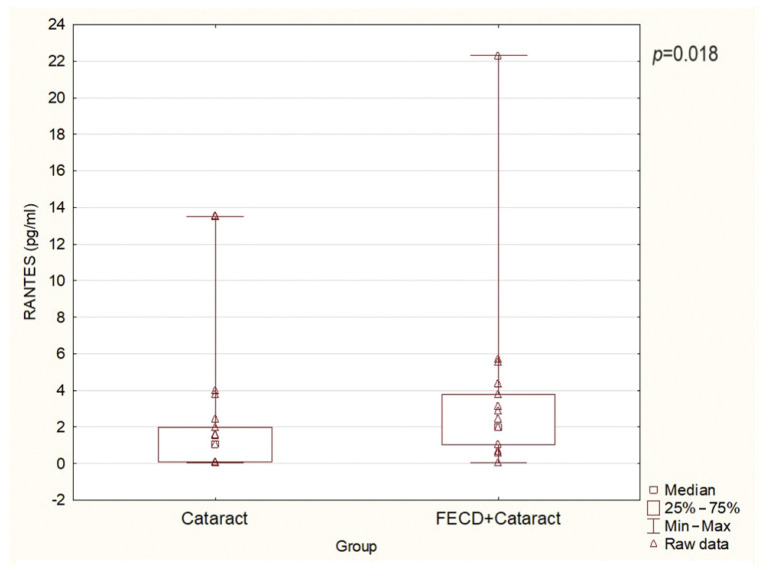
The levels of RANTES (CCL5) in AH in the study groups (*p* = 0.018).

**Figure 2:**
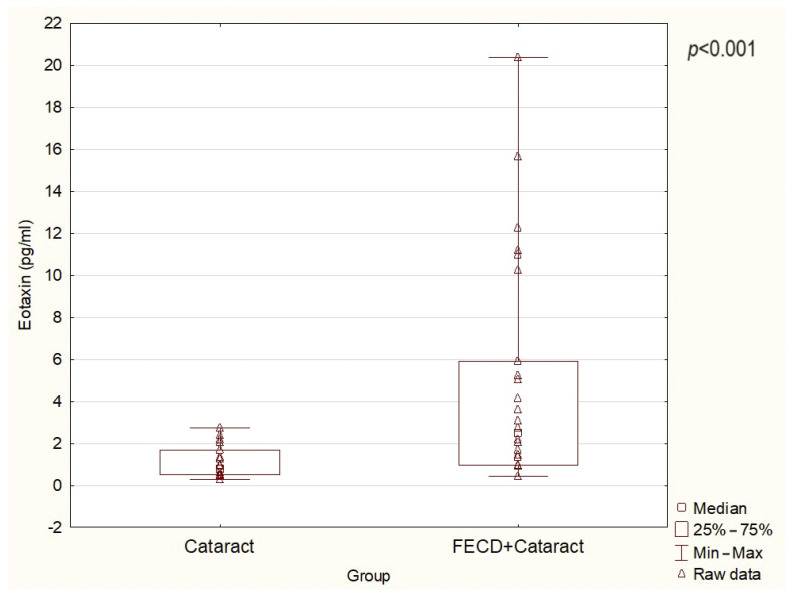
The levels of eotaxin (CCL11) in AH in the study groups (*p* = 0.001).

**Figure 3:**
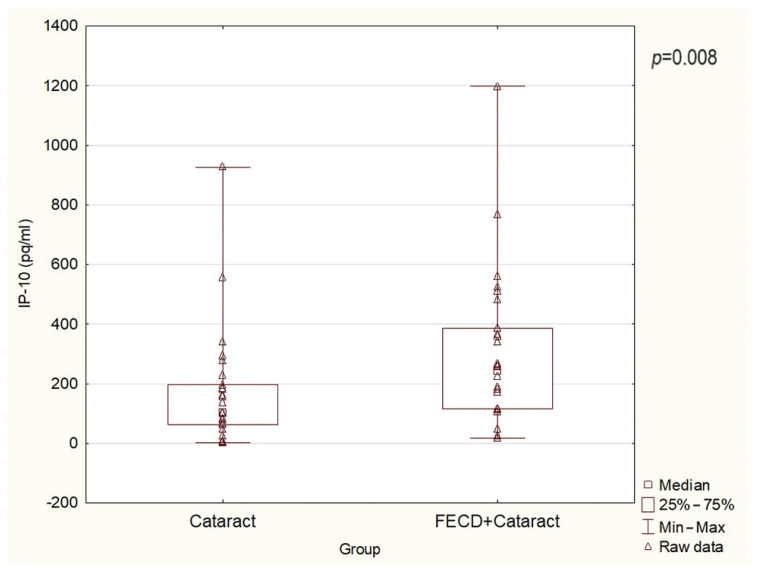
The levels of IP-10 (CXCL10) in AH in the study groups (*p* = 0.008).

Figure 1, Figure 2 and Figure 3 present the level of RANTES, eotaxin, and IP-10, respectively. Table 2 presents the levels of all chemokines assessed in the present study. The mean AH levels of RANTES, eotaxin, and IP-10 in the FECD + cataract group were significantly higher than in the cataract/control group (*p* = 0.04, 0.001, 0.01, respectively). There were no differences in the levels of MIP-1α, MCP-1, and MIP-1β between the groups (Table 2).

Table 3 presents the levels of growth factors in AH in the studied groups. There were no statistically significant differences in the levels of studied growth factors (FGF-basic, GM-CSF, PGDF-BB, G-CSF and VEGF) between the groups.

There were no statistically significant differences in the levels of studied growth factors (FGF-basic, GM-CSF, PGDF-BB, G-CSF, and VEGF) between the groups. However, the level of G-CSF and VEGF were higher in FECD + Cataract group than in Cataract/control group (respectively 6.60 vs. 1.44 for G-CSF, and 15.62 vs. 7.25 for VEGF).

## 3. Discussion

The eye is an organ of vision with unique immunological features that result from its anatomy, physiology, and the presence of specific elements, ensuring homeostasis of the eyeball. The anatomical and physiological status of the eyeball influences the creation of a unique barrier that determines the formation of the immunological privilege. The proper functioning of the cornea and lens is ensured by AH with anti-inflammatory and immunosuppressive properties. Moreover, the AH removes harmful metabolic products and supplies nutrients [11,12]. The influence of a variety of triggering factors, such as environmental factors, surgeries, or comorbidities may cause an influx of pro-inflammatory cytokines into the AH and, consequently, may contribute to the development of eye diseases.

The coexistence of cataract and FECD often observed in our medical practice, and the resulting problems related to the implementation of appropriate procedures during cataract surgery, prompted us to evaluate potential biomarkers that could allow us to differentiate patients with FECD associated with cataracts from those suffering from cataracts only. An important criterium was to implement these potential biomarkers before planning cataract surgery in order to establish endothelium-sparing operating procedures. Because of recent advancements of immunology in eye diseases, our research focused on inflammatory chemokines and growth factors.

Chemokines, discovered in the 1990s, can impact various metabolic and immunological processes, including intracellular regulation, immune responses, leukocyte chemotaxis stimulation, adhesion molecule activation, leukocyte activation and differentiation, cell proliferation and apoptosis regulation, angiogenesis, embryogenesis, and organogenesis. They also play a role in the pathogenesis of inflammatory, autoimmune, and proliferative diseases [13]. Similar to other cytokines, their altered levels in various disease states can serve as targets for research, identifying specific chemokines as prognostic and diagnostic markers. Moreover, they can serve as promising therapeutic targets for medical interventions. The CC (beta) chemokines represent the largest chemokine group, consisting of factors with diverse functions. This group includes MCP-1, MIP-1 alpha, MIP-1 beta, RANTES, and eotaxin, which were the subject of this study. All these chemokines are typical chemoattractants, i.e., molecules that induce directed cell migration in response to environmental signals in cells capable of movement. The intensity of the response to the chemoattractant depends on the gradient of its concentration in the extracellular medium [14]. The synthesis of chemokines is subject to multi-level regulation, and their expression is stimulated by various signals, including hypoxia, bacterial degradation products, oxidative stress, thrombin, and proinflammatory cytokines like tumor necrosis factor alpha (TNF-α), IL-1, Interferon-gamma (INF-γ), and interleukin 6 (IL-6) [15].

Numerous studies have demonstrated the involvement of CC chemokines in the pathogenesis of allergic diseases, HIV-1 infection, rheumatoid arthritis, multiple sclerosis, transplant rejection, and asthma [15,16]. However, only a few studies have focused on the role of chemotactic factors in the development and progression of eye diseases such as dry eye [17], macular degeneration [18], keratoconus [19] and FECD [20]. These studies determined chemokine levels in various body fluids (e.g., tears, serum, and aqueous humor) and various eye conditions, indicating the involvement of cytokines, including chemokines, and growth factors in eye disease development.

Regarding chemokines in which AH levels differed significantly in the FECD + cataract group versus the cataract group, eotaxin has the strongest chemoattractive properties acting on eosinophils. Eotaxin is involved in eosinophilic inflammatory diseases, such as atopic dermatitis, allergic rhinitis, asthma, and parasitic infections [15]. IP-10 belongs to the CXC group of chemokines. It stimulates monocytes, natural killer cells, migration of T lymphocytes to endothelial cells, and modulation of the expression of adhesion molecules. It is involved in the pathogenesis of rheumatoid arthritis, multiple sclerosis, transplant rejection, and asthma [15]. RANTES has chemotactic activity for monocytes, T helper lymphocytes, and eosinophils. It causes the release of histamine from basophils and activates eosinophils. RANTES has been shown to participate in the pathogenesis of allergic diseases, viral infections, rheumatoid arthritis, multiple sclerosis, transplant rejection, and asthma [15]. In addition, MCP-1 levels showed a tendency to be elevated; however, these differences were not sufficient enough to be considered statistically significant. MCP-1 has chemotactic activity for monocytes and basophils. Its elevated levels have been demonstrated in rheumatoid and psoriatic arthritis, atherosclerosis, multiple sclerosis, transplant rejection, allergic diseases, and asthma [21].

It should be stressed that these parameters were determined by studying eyes without corneal transplantation and before cataract surgery, as some authors have linked elevated cytokine levels only to the postoperative state [22,23]. The observed significantly higher levels of RANTES, eotaxin, and IP-10, along with non-significantly elevated levels of MCP-1 promote the development of inflammatory responses in FECD eyes. As chemotactic factors, those cytokines could be involved in an influx of inflammatory cells into the AH of FECD eyes, which could intensify inflammatory effects, and contribute to both accelerated cataract development and exacerbated corneal changes associated with FECD. Moreover, the observed changes in the AH levels of these chemokines suggest disruption of ocular immune privilege in FECD. The mechanisms of elevated AH levels of RANTES, eotaxin, and IP-10 in FECD are unknown. While the expression of inflammatory chemokines is usually controlled by NF-kB [24,25], the induction of these chemokines was highly selected in the present study and not accompanied by more robust inflammatory responses typically associated with the activation of this transcription factor.

Consistent with the findings of this study, altered levels of these cytokines have also been shown to be associated with eye disorders. Haozhe et al. in their study on tear cytokine levels in meibomian gland dysfunction-related dry eye found that eotaxin and CXCL10 decreased after light therapy, resulting in reducing inflammation [17]. Miyagawa et al. indicated that eotaxin-2 levels in tears may be a suitable biomarker associated with severe allergic conjunctival disease [26]. Mo et al. determined serum cytokine levels in subjects with different stages of age-related macular degeneration (AMD), and found that eotaxin and IP-10 may be early biomarkers in AMD. They also hypothesized that a relative balance between levels of IP-10 and eotaxin may be critical in regulating the neovascular response [18]. Shoji et al. found that the mRNA expression and protein levels of the eotaxin subfamily members on the ocular surface are critical biomarkers when investigating the pathophysiology of eosinophilic inflammation and the effect of antiallergic treatment in patients with vernal keratoconjunctivitis (VKC) [27]. Matthaei et al. presented a study on the expression of epithelial–mesenchymal-transition (EMT)-related cytokines (TGF-b1, TGF-b2, TGF-b3, MCP-1, BFGF, TNF-a, and IL-1b) in the AH of phakic (FECDph) and pseudophakic FECD (FECDpsph) eyes in comparison to a cataract group. Because no differences in protein levels were detected in FECDph eyes compared to cataract eyes, the authors concluded that these cytokines did not play a major role in FECD pathogenesis. However, an elevation of TGF-b1, TGF-b2, and MCP-1 levels in post-surgery FECDpsph eyes confirmed that cataract surgery can result in long-term alterations of the intraocular microenvironment, and altered cytokine levels may be involved in corneal decompensation after cataract surgery [22]. Furthermore, De Roo with coauthors reported that MCP-1 and IL-8 increased in response to cataract surgery, and that the TGF-b family of cytokines likely plays a role in FECD [23]. The observations made by Matthaei et al. and De Roo differ from ours. The authors of the aforementioned papers believe that elevated cytokine levels are a result of the surgery performed (corneal transplantation or cataract surgery, respectively), which disrupts the immunology of the eye, rather than the development of the disease itself. In our study, we included individuals in the first stage of FECD development who had not undergone prior surgery. Hence, higher chemokine levels in eyes with FECD + cataract compared to eyes with only cataract indicate that these parameters may be predictive factors in the development of FECD.

In addition to chemokine levels, our study evaluated the levels of several growth factors in the AH of FECD and/or cataract eyes. There was a strong tendency for the AH levels of VEGF and G-CSF in the FECD plus cataract group to be higher as compared to the cataract-only group; however, these differences were not statistically significant. In contrast, there were no differences in the levels of FGF-basic, GM-CSF, and PDGF-bb levels. Alterations of growth factors have been shown in several eye disorders. Fischenko et al. found that GM-CSF (in addition to MCP-1 and MIP-1β levels) were significantly higher in FECD eyes when compared to individuals with healthy eyes, the control group. The authors concluded that FECD was associated with the disruption of ocular immune privilege that leads to chronic local inflammation, which in turn causes remodeling of the corneal tissues resulting in fibrosis [20].

The differences between the aforementioned studies and ours may stem from the fact that the patients in our study were in the first stage of FECD, whereas in the other studies, they had advanced disease.

Wang and Tao observed that expression of VEGF and bFGF in the AH of Fuchs uveitis syndrome (FUS) patients was significantly higher than in senile cataract eyes. Moreover, these changes were associated with elevated levels of IL-6 and IL-8, and were positively associated with the severity of posterior subcapsular cataract [28]. These results imply that an inflammation mechanism may be involved in the early development of cataract in FUS.

The differences between our results may be attributed to a distinct disease entity. Wang and Tao conducted a study on patients with Fuchs uveitis syndrome, while we focused on patients with Fuchs endothelial dystrophy.

In our study, the median VEGF levels increased over 100% in the FECD + cataract group as compared to the cataract/control group. While these changes were not significantly different, due to high variability between studied subjects, they may suggest an increase in permeability of eye capillaries, which can contribute to the development of FECD-associated pathology by stimulation of inflammation, edema formation, and trafficking of inflammatory cells. Indeed, VEGF represents a growth factor with important mitogenic and anti-apoptotic effects on endothelial cells, increasing vascular permeability, promoting cell migration, and remodeling of the extracellular matrix. Due to these effects, VEGF actively contributes to regulating the normal and pathological angiogenic processes. Both the inflammatory processes and immune reactions promote the expression of VEGF and its receptors. VEGF is a strong mitotic and chemotactic factor for endothelial cells, which stimulates the formation of new vessels and increases endothelial permeability [29].

### Study Limitations

The obtained results are innovative but require further research on a larger group of patients. The relatively small size of the study groups employed in the present pilot study resulted from the fact that FECD is a relatively rare ocular disease. Therefore, a multi-site clinical research study is recommended for establishing reliable biomarkers of FECD.

## 4. Materials and Methods

The study was performed from 2021 to 2023 in accordance with the standards of the Declaration of Helsinki. The procedures were approved by the Bioethical Committee of the Medical University of Silesia (PCN/CBN/0022/KB1/84/21; 15 June 2021). All potential risks were explained to patients during recruitment, and written informed consent to participate in the study and publish the research results was obtained from all patients. To avoid bias, all samples have been anonymized and numbered. Patients’ data have been protected by a RODO statement (General Data Protection Regulation).

### 4.1. Study Design and the Participants

**Figure 4:**
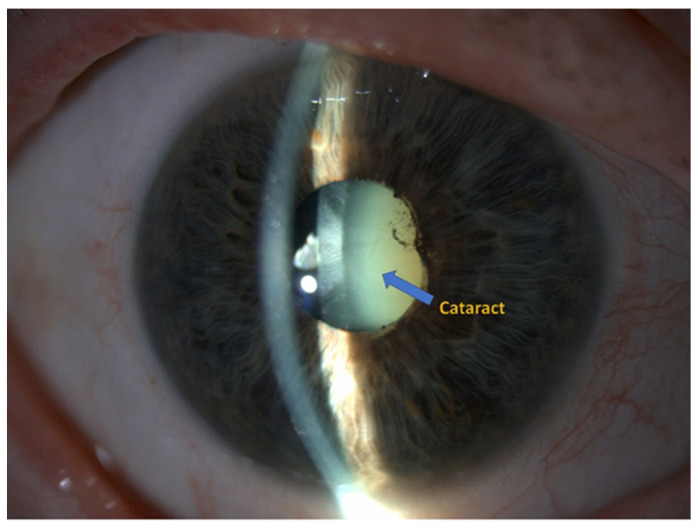
Cataract [Department of Ophthalmology, Faculty of Medical Sciences in Zabrze, Medical University of Silesia, Katowice, Poland].

**Figure 5:**
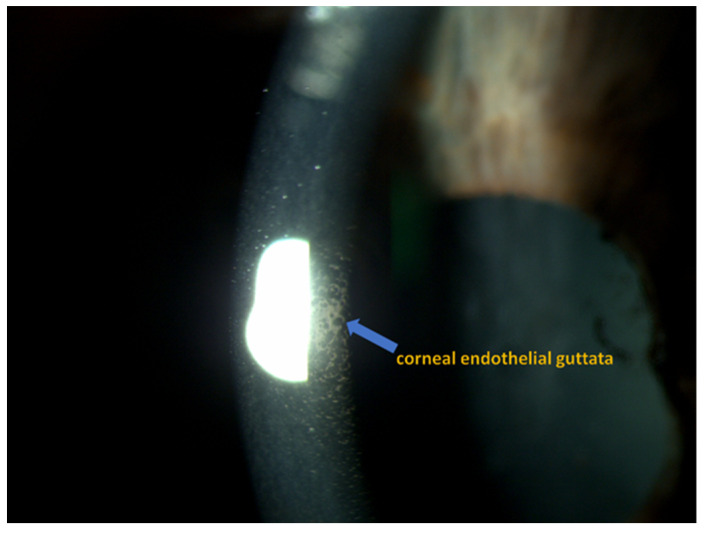
Corneal endothelium in FECD [Department of Ophthalmology, Faculty of Medical Sciences in Zabrze, Medical University of Silesia, Katowice, Poland].

The clinical part of the study was conducted in the Department of Ophthalmology, and the analytic part in the Department of Microbiology and Immunology at the Medical University of Silesia in Katowice, Poland. The study recruited 52 patients in total (32 females and 20 males; age 71.77 ± 7.59 years) with non-significant (*p* > 0.05) distribution between the groups. Inclusion criteria were as follows: diagnosis of FECD (Figure 4), diagnosis of cataract (Figure 5), individuals over 18 years of age, and written informed consent to participate in the study and publish the research results. Exclusion criteria included pregnancy, cancer, eye injuries, glaucoma, inflammatory conditions of the uveal membrane of the eye, systemic steroid therapy, and failure of patients to provide written consent.

**Figure 6:**
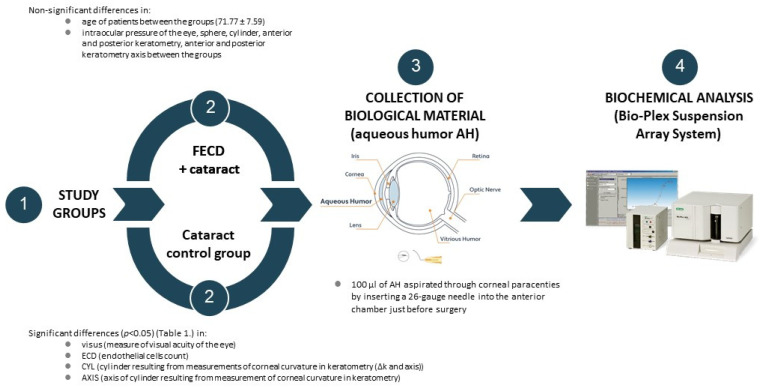
Study design. Description of the study groups, sample collection, and analysis.

The patients were divided into two study groups: the FECD + Cataract group (n = 26) and the Cataract/control group (n = 26). Because obtaining aqueous humor from healthy patients was not recommended by the Bioethics Committee, cataract patients were utilized as the control group. Thus, healthy patients could not be included as controls in the study. The diagnosis of FECD was based on a comprehensive examination and recognized diagnostic criteria [30,31]. Imaging assessments were performed for each patient prior to cataract surgery to assess the status of the disease. These assessments included optical coherence tomography (OCT) imaging in the SS CASIA, SD REVO OCT, and slit-lamp examination with photographic documentation. The study design is presented in Figure 6.

### 4.2. Sample Collection

To collect samples of AH, the eyelids and skin around the eyes were wiped with disinfectant, and 100 µL of AH was aspirated through corneal paracentesis by inserting a 26-gauge needle into the anterior chamber just before surgery. All samples were collected into sterile tubes and immediately frozen in −82 °C until the future analysis. Collecting AH did not adversely affect the cataract surgery and was not associated with additional complications.

### 4.3. Biochemical Assessment

The biochemical analyses were carried out in blinded samples by experienced scientific personnel of the Department of Microbiology and Immunology at Silesian Medical University in Zabrze. All procedures followed good laboratory practice. The assessments were performed using the Bio-Plex 200 System from Bio-Rad and the Bio-Plex Pro Human Cytokine Panel 27-Plex (#M500KCAF0Y). The Bio-Plex Suspension Array System included fluorescently labelled microspheres and instrumentation licensed to Bio-Rad Laboratories, Inc. by the Luminex Corporation (Austin, TX, USA). Thirty µL of each AH sample was used per analysis. Standard curves were generated using the reference standards supplied with the kits and used to determine respective analyte concentrations for each sample. Minimum detection thresholds of individual analytes were MCP-1 0.2 pg/mL, MIP-1alpha 0.05 pg/mL, MIP-1beta 0.05 pg/mL, RANTES 0.02 pg/mL, Eotaxin 0.1 pg/mL, IP-10 1.0 pg/mL, IFN-γ 0.1 pg/mL, FGF basic 0.5 pg/mL, G-CSF 0.05 pg/mL, GM-CSF 0.02 pg/mL, PDGF-bb 2.0 pg/mL, and VEGF 0.5 pg/mL.

### 4.4. Statistical Analysis

Statistica v. 13.3 (Tibco, Palo Alto, CA, USA) software was used for statistical analysis. The Shapiro–Wilks test was used to assess normality, evaluating the distribution of each relevant variable. For comparative analysis, the U Mann–Whitney test was applied to variables with non-parametric distribution, while the parametric *t*-test was used for parametric variables like age. The Chi-squared test was utilized to examine gender distribution differences between the groups. Statistical significance was assumed at a *p*-value of <0.05.

## 5. Conclusions

In this study, we observed higher levels of RANTES, eotaxin, and IP-10 in the AH of patients with FECD and cataract as compared to patients who suffered from cataract only. These chemokines may play a role in FECD development and, importantly, could be considered as potential biomarkers of this disease that may allow differentiation of FECD from cataracts. The finding of elevated biochemical marker concentrations in a patient without a clinical diagnosis may be an indication for increased observation of the other eye for the development of FECD.

## Figures and Tables

**Table 1:** Statistically significant key ophthalmological variables measured in patients with a specific eye condition with *p*-values between the groups.

	FECD + Cataract				Cataract/Control		
Variable	Median	Minimum	Maximum	*p* Value	Median	Minimum	Maximum
visus	0.10	0.02	0.60	0.001	0.40	0.02	1.00
ECD	1282.00	886.0	1395.00	0.01	2452.00	1908.00	3331.00
CYL	−1.28	−18.10	−0.46	0.001	−0.56	−4.09	0.00
AXIS	118.00	10.00	179.00	0.03	32.00	0.00	177.00

Visus—measure of visual acuity of the eye; ECD—endothelial cells count; CYL—cylinder resulting from measurement of corneal curvature in keratometry (Δk and axis); AXIS—axis of cylinder resulting from measurement of corneal curvature in keratometry.

**Table 2:** Chemokine levels determined in aqueous humor of patients’ eyes in the studied groups.

	Group (1) FECD + Cataract				Group (2) Cataract/Control		
Variable in [pg/mL]	Median	Min	Max	*p*-Value	Median	Min	Max
MCP-1 (CCL2)	462.36	60.37	6239.71	0.284	301.26	0.30	4025.51
MIP-1α (CCL3)	0.52	0.08	4.41	0.545	0.52	0.01	16.69
MIP-1β (CCL4)	6.85	2.09	130.06	0.558	5.22	0.28	359.61
RANTES (CCL5)	1.98	0.03	22.32	0.018	1.04	0.04	13.51
Eotaxin (CCL11)	2.46	0.42	20.37	<0.001	0.77	0.27	2.74
IP-10 (CXCL10)	239.99	16.73	1197.18	0.008	100.98	1.40	925.68
IFN-γ	4.00	0.97	15.31	0.22	2.66	0.13	15.08

MCP-1 (CCL2)—monocyte chemotactic protein 1, MIP-1α (CCL3)—macrophage inflammatory protein 1 alpha, MIP-1β (CCL4)—macrophage inflammatory protein 1 beta, RANTES (CCL5)—regulated on activation, normal T-cell expressed and secreted, eotaxin (CCL11)—eosinophil-specific chemoattractant, IP-10 (CXCL10)—interferon-gamma (IFN-gamma)-inducible protein-10, IFN-γ—interferon-gamma.

**Table 3:** Growth factor levels determined in aqueous humor of patients’ eyes in the studied groups.

	Group (1) FECD + Cataract				Group (2) Cataract/Control		
Variable in [pg/mL]	Median	Min	Max	*p*-Value	Median	Min	Max
FGF basic	5.87	3.52	651.72	0.94	5.87	0.72	13.69
G-CSF	6.60	0.41	76.99	0.09	1.44	0.07	124.56
GM-CSF	0.28	0.17	1.91	0.62	0.29	0.02	1.18
PDGF-BB	4.71	2.79	42.27	0.59	4.71	4.19	11.00
VEGF	15.62	3.34	132.84	0.26	7.25	0.24	40.77

FGF basic—fibroblast growth factor-2, G-CSF—granulocyte colony stimulating factor, GM-CSF—granulocyte macrophage colony stimulating factor, PDGF-BB—platelet-derived growth factor BB, VEGF—vascular endothelial growth factor.

## Data Availability

The database of aggregated statistics ready for analysis is stored in a secure and password-protected repository on the server of the Medical University of Silesia. The data were anonymized. Completely non-identifiable records are available to interested persons/organizations upon request from the corresponding author at fiolkarafal@gmail.com.
